# Editorial: Dishonest Behavior, from Theory to Practice

**DOI:** 10.3389/fpsyg.2016.01521

**Published:** 2016-09-30

**Authors:** Shahar Ayal, Guy Hochman, Dan Ariely

**Affiliations:** ^1^Interdisciplinary Center Herzliya, Baruch Ivcher School of PsychologyHerzliya, Israel; ^2^Center for Advanced Hindsight, Social Science Research Institute, Duke UniversityDurham, NC, USA

**Keywords:** dishonesty, moral self, ethical dissonance, cross cultural, interventions

The rapidly growing field of behavioral ethics has shown that dishonest acts are highly prevalent in all walks of life, from corruption among politicians to flagrant cases of doping in sports, to everyday slips, and misdemeanors by ordinary people who nevertheless perceive themselves as highly moral. For instance, managers exaggerate travel expenses, consumers engage in wardrobing, citizens evade taxes, or download illegal music. When considered cumulatively, these seemingly innocuous and ordinary unethical behaviors cause considerable societal damage and add up to billions of dollars annually (Ariely, 2012).

Recent works in the behavioral ethics field have made tremendous advances in understanding the roots of dishonesty and characterizing the contextual and social factors that promote or hinder it. For example, one of the main insights is that people value morality and try to resist the temptation to act dishonestly (Aquino and Reed, 2002; Bazerman and Tenbrunsel, 2011). Investigations of misconduct in real life and in laboratory experiments indicate that while most people act dishonestly in everyday life, their dishonest acts are usually far below the maximum possible (Gneezy, 2005; Mazar et al., 2008; Shalvi et al., 2011). According to the Self-Maintenance model of dishonesty, this is due to *ethical dissonance* (Ayal and Gino, 2011; Barkan et al., 2012), a psychological tension which stems from the conflict between the desire to benefit from unethical behavior and the motivation to maintain a positive moral image (Barkan et al., 2015; Hochman et al., 2016).

The current research topic aims to utilize these lines of work to shift research in behavioral ethics from a descriptive approach to a more prescriptive and applicable one, thus advancing theoretical knowledge and making it possible to implement the findings to design and test practical interventions to promote ethical conduct among individuals in their day to day lives.

The first section explores the processes underlying dishonesty and highlights the interplay between moral self-image (MSI) and dishonesty. The second section sheds more light on contextual factors that promote or hinder dishonesty, with special attention to the perceived reasons and consequences of behavior. The last two sections emphasize the role of social and cultural norms both in the form of dishonesty as well as in effective interventions to reduce it.

## Moral self-image and dishonesty

Several works examine the interplay between peoples' MSI and dishonest behavior. Jordan et al. aim to capture the fluctuations and malleability of the moral self in that people perceive themselves as highly moral, but engage routinely in unethical behavior. The paper defines the construct of MSI and presents an assessment questionnaire for moral self-perception in a current state. Liang et al. test how self-esteem affects the intention to engage in corrupt behavior. They show that increased self-esteem causes a low level of materialism, which in turn decreases corrupt intention.

In a similar vein, Ding et al. delve deeper into the dynamics of MSI by examining the functions of guilt and moral identity in motivating prosocial behavior. They show that the link between acting immorally and compensatory behavior is mediated by guilt and moderated by moral identity (for related “moral accounting” models see Sachdeva et al., 2009; Gneezy et al., 2014). Merzel et al. show that people who acted dishonestly in the past are ready to suffer a future loss rather than admitting, even implicitly, that they lied. Chance et al. however show that this self-deception will decay if individuals are exposed to unbiased feedback about their true ability, but can be quickly revived if they get a new opportunity to cheat.

The last paper in this section (Burgoon) challenges the fundamental assumption that lying requires more effort than truth-telling. The paper discusses communication factors that may moderate the cognitive effort associated with producing deceptive messages.

## Contextual factors that promote or inhibit dishonesty

One way to resolve the tension between dishonest behavior and MSI is to creatively interpret an incriminating behavior as an honest or acceptable one (Mazar et al., 2008; Barkan et al., 2015). As a result, the magnitude of dishonesty is highly sensitive to contextual factors that affect our ability to justify unethicality (Shalvi et al., 2015; Hochman et al., 2016). Applying these insights, Rilke et al. show that self-reporting work hours dishonestly can be reduced by moving from a one-by-one to an all-at-once reporting policy. Van Der Zee et al. reported that negative emotional responses in an online settings (i.e., rejection) leads to increased dishonest behavior. By contrast, Lang et al. found that religious music can be used as a subtle cue associated with moral standards to curb dishonest behavior, but this mainly affects religious participants. Finally, the magnitude of dishonesty is also sensitive to the perceived identity of its victims. Amir et al. suggested that people are more willing to cheat groups than individuals, but only when the harm to the group is stated in global terms. In this context the lack of information about the harm caused to each individual can be used as a pretext for cheating.

## Cross-cultural differences in dishonest behavior

Social and cultural norms play a key role in shaping moral behavior (e.g., Cialdini, 1993; Haidt and Joseph, 2004). In a study on cross-cultural differences in tax evasion between Italy and Sweden, Andrighetto et al. find that even though average tax compliance is similar in both countries, Italians were much more likely to fudge their income and Swedes were more likely to be completely honest or dishonest. Mann et al. found that legal sanctions and internal factors designed to deter minor, non-violent crimes have similar effects on different dishonest acts across five distinct cultures. More specifically, the results indicated that across countries, internal sanctions had the strongest deterrent effects on crime. However, the deterrent effects of legal sanctions were weaker and varied across countries.

## From theory to practice: research using real-world situations and field data

This research topic emphasized the applicability of unethical decision-making research in the real world. Moore and Pierce combines experimental and field data to examine how authorities penalize transgressors when the social context of the transgression elicits expectations of leniency. A surprising finding suggests that expectations of leniency (e.g., when the transgressor is caught on his birthday) appear to elicit psychological reactance and lead to stricter punishment.

The last two papers in this topic directly investigate the academic community itself, and speculate how to foster high ethical standards to improve scientific integrity. Rajah-Kanagasabai and Roberts show that engagement in research-related misconduct and questionable research practices is affected by attitudes, subjective, and descriptive norms about dishonesty, and mediated by justifications and behavioral intentions. Similarly, Schoenherr discusses potential practices (e.g., incentivizing quality rather than quantity of research) that may solve the problem of inappropriate authorship and encourage ethical behavior within the research community.

## Conclusion

Research in the rapidly grown field of behavioral ethics suggests that public policies and interventions that are based on empirical research may encourage people to live according to higher ethical standards (Ariely, 2012; Ayal et al., 2015). The current research topic presents a wide array of research that contributes directly to this laudable goal. Taking together, these articles suggest that dishonest behavior in different forms and cultures share similar underlying processes. Thus, effective solutions to curb dishonesty and promote moral behavior in different domains (and across cultures) should be composed of the same psychological building blocks.

## Author contributions

SA, GH, and DA equally contributed to the research topic and for the writing of this Editorial.

### Conflict of interest statement

The authors declare that the research was conducted in the absence of any commercial or financial relationships that could be construed as a potential conflict of interest.
